# COVID-19 Mortality In Adult Congregate Living Settings Across Five U.S. States

**DOI:** 10.1093/geroni/igab046.2010

**Published:** 2021-12-17

**Authors:** David Rein, Lindsey Shapiro, Mairin Mancino, Caroline Pearson

**Affiliations:** 1 NORC, Atlanta, Georgia, United States; 2 NORC, NORC, Chicago, Illinois, United States; 3 NORC, NORC, Maryland, United States

## Abstract

The magnitude of COVID-19 mortality in adult congregate living settings other than nursing homes (NH) is unknown. To address this, we created an individual property level dataset for five U.S. states (Colorado, Connecticut, Florida, Georgia, and Pennsylvania) using multiple public and private sources. The data included information on each observation’s state and county, level of care (LOC), the estimated number of residents, COVID-19 deaths through December 31, 2020, and county-level cases of COVID-19 per 100,000. We restricted our sample to market grade properties with 25+ residents, for which we able to estimate resident and LOC information. We defined LOC as County, non-congregate (CN), Independent Living (IL), Assisted Living (AL), Memory Care (MC), and Nursing Home (NH). We used multilevel, multivariable logistic regression models to estimate the expected death rate for each LOC controlling for differences in reported COVID-19 infections and county and state reporting differences. We identified 3,059 properties that met our inclusion criteria (69 CN, 477 IL, 1,118 AL, 179 MC, and 1,216 NH). We estimated deaths per 1,000 persons of 4.4 (95% CI: 4.0-4.8) for CN, 4.4 (3.9-4.9) for IL, 16.2 (14.7-17.9) for AL, 50.3 (44.4-56.8) for MC, and 32.0 (29.3-34.9) for NH. The order of death rate severity was the same in each state, except MC in Georgia. Additional research is needed to evaluate whether death rate differences resulted from congregate living risks, from COVID mortality risk factors at each LOC, or a combination of factors.

